# Does mindfulness help to overcome stereotype threat in mental rotation in younger and older adolescents?

**DOI:** 10.1007/s00426-022-01666-y

**Published:** 2022-03-18

**Authors:** Martina Rahe, Petra Jansen

**Affiliations:** 1 University of Koblenz-Landau, Universitaetsstrasse 1, 56070 Koblenz, Germany; 2 University of Regensburg, Universitaetsstrasse 31, 93053 Regensburg, Germany

## Abstract

We investigated gender differences in mental rotation performance in younger and older adolescents and effects of stereotype threat activation and a short mindfulness induction. Two hundred fifty younger adolescents from grades 5, 6, and 7 (119 boys) and 152 older adolescents from grades 10, 11, and 12 (80 boys) were divided into four groups with or without a mindfulness induction and with or without stereotype threat activation. All participants solved a mental rotation test and filled out a questionnaire about their gender stereotype beliefs and perceived abilities of masculine and feminine activities. Results illustrate that older adolescents outperformed younger adolescents, and gender differences in favor of males appeared only in the older age group. Independent of gender, the mindfulness induction had a significantly positive effect on adolescents’ mental rotation performance that was significant only in the older age group. No effect of the stereotype activation was found. For gender stereotype beliefs and perceived abilities of gendered activities, the mindfulness intervention enhanced male stereotype beliefs and participants’ perceived ability of masculine activities. A short mindfulness induction seems to have an enhancing effect on a subsequently performed stereotypically masculine cognitive task and consequently on adolescents’ male stereotype beliefs and their perceived ability in masculine activities.

## Introduction

Mental rotation describes the ability to imagine an object turned around in mind (Shepard & Metzler, 1971). According to a classification of Uttal et al. (2013), it can be classified as an intrinsic-dynamic spatial ability, which is, for example, important for mathematical abilities (Mix et al., 2016). Thereby it seems to be plausible that different components of the mental rotation task (e.g., encoding of stimuli features, imagined angular rotations, and fast stimulus matching) are related to various components of mathematical tasks as, for example, the number line estimation (symbolic number sense, original magnitude sense, and line subdivision) (Young et al., 2018). Furthermore, an increased mental rotation performance is related to the attendance of a high school focusing on school subjects such as chemistry, design, or biology (Moè, 2016b). Hence, mental rotation is a crucial ability, e.g., in education, and is widely investigated with either chronometric or psychometric mental rotation tests. Whereas in chronometric (computer-based) mental rotation tests, two rotated objects (mirror-reversed or not) are presented, and the participants have to decide if both objects are identical or not, in psychometric (paper–pencil) tests, each task contains of one target item on the left side and four sample stimuli on the right (Peters et al., 1995).

Spatial abilities and the ability of mental rotation are malleable and modifiable (Uttal et al., 2013). A meta-analysis showed that training with video games, courses, or spatial tasks could enhance the performance in spatial tasks. Moreover, the positive effects of these training also transferred to other spatial tasks (Uttal et al., 2013). The ability of mental rotation may be influenced by experience, e.g., in spatial activities (Baenninger & Newcombe, 1989), sports (Jansen & Pietsch, 2010), and music (Pietsch & Jansen, 2012).

Especially in psychometric mental rotation tests, meta-analyses found gender differences favoring boys and males in childhood (Lauer et al., 2019), adolescence (Lauer et al., 2019; Voyer et al., 1995), and adulthood (Voyer et al., 1995). In this study, we examine whether these gender differences can be influenced by stereotype threat manipulation and mindfulness induction.

### Gender differences in mental rotation during childhood and adolescence

The gender difference in mental rotation is the largest documented cognitive gender difference (Halpern, 1989). As mentioned above, the existence of this effect is widely accepted—at least with the beginning and after puberty and for paper–pencil mental rotation tests (Voyer et al., 1995). Gender differences favoring boys were detected in children as young as ten years with different types of stimuli (cube figures, letters, and animal drawings) (Neuburger et al., 2011). This advantage of boys might be more prominent with older fourth graders (Titze et al., 2010) and might also appear in adolescence (Titze et al., 2011). However, the stability of gender differences, especially measured with the MRT (mental rotation test) during adolescence, is not agreed upon. In a study with 144 adolescents between 12 and 17 years (89 boys and 55 girls), no gender difference could be detected in this specific mental rotation task (Jansen et al., 2018). In contrast, Rahe and Quaiser-Pohl (2019) showed that gender differences in the MRT were not significant in a younger age group (up to 15 years), but appeared in an older age group (from 15–20 years). Therefore, we tested younger adolescents in grades 5–7 (10 and 14 years old) and older adolescents in grades 10–12 (15–18 years old) to examine how gender differences and beliefs change between these two critical educational time points.

If gender differences appear, biological and social–cultural explanations are often discussed to explain gender differences in beginning adolescence. One biological explanation is the beginning of the pubertal-hormonal changes. Social–cultural explanations might be different socializations of girls and boys (Baenninger & Newcombe, 1989). Preferring spatial toys in childhood (Moè et al., 2018), choosing a STEM-subject over a non-STEM-subject (Moè et al., 2018), the preference for soccer over dancing in girls (Pietsch et al., 2019), attending a single-sex school (Titze et al., 2011), or exposure to mothers’ spatial language use (Ralph et al., 2021) can enhance mental rotation performance. Other social–cultural explanations might be the self-concept as well as the activation of a stereotype threat (Steele & Aronson, 1995), which is the focus of this study.

### Stereotype threat effects in mental rotation tasks

Gender stereotypes are social phenomena which describe the beliefs about the characteristics of females and males. Until now, several studies have demonstrated the influence of gender stereotype activation on the mental rotation performance in adults (e.g., Moè, 2009; Moè & Pazzaglia, 2006) and in children (Neuburger et al., 2013). Stereotype lift describes that someone thinks that the *own* group performs better in a particular topic (Walton & Cohen, 2003). It has been shown that females’ results in spatial tasks were better when those tasks were manipulated with a stereotype lift (Doyle & Voyer, 2016).

Stereotype threat is defined as “the risk of confirming, as self-characteristic, a negative stereotype about one’s group” (Steele & Aronson, 1995, p. 797) and can reduce participants’ performances in cognitive tests by triggering a disruptive mental load (Croizet et al., 2004). Many studies have investigated the effect of a stereotype threat condition on the performance in a mental rotation task and possible ways to diminish these adverse effects (e.g., Hausmann, 2014; Martens et al., 2006). Results showed that female art students were more vulnerable to a threat condition than female science students (Hausmann, 2014). Furthermore, effects of a stereotype threat activation on the performance in a mental rotation task were found in 10 years old children (Neuburger et al., 2015). On the other hand, it has been shown that not all women suffer from stereotype threat activation in mental rotation. In a study, this was only visible in women with a feminine gender role orientation (Tempel & Neumann, 2016). Moreover, making females aware of their positive characteristics and values before solving the test could curb negative effects of a stereotype threat condition (Martens et al., 2006). Hence, the effects of stereotype threat activation on the mental rotation performance are not consistent and may be mitigated by stronger beliefs about one’s ability. One possible factor in enhancing adolescents’ beliefs could be the training of spatial activities.

A spatial training for women with mental rotation tasks (Meneghetti et al., 2016) or the training of women’s motivation (Moè, 2016a) can help to increase their performance. Moreover, motivational and strategic training—or the combination of both—can enhance the mental rotation performance of high school students (Moè, 2021). Furthermore, training with spatial computer games (Neubauer et al., 2010) or the work with spatial skill-building material in school (Sorby, 2009) could enhance females’ performance in a mental rotation test more than males’. Additionally, testing participants in a group composition with same or mixed-gender groups can affect gender stereotypes (Hirnstein et al., 2014) and the performance in a mental rotation task (Moè, 2018). In conclusion, different training methods are suitable to increase the performance in a mental rotation task and could curb the adverse effects of stereotype threat to females as well as personality characteristics or talents or self-affirmation. One main question of the present study is whether a stereotype threat can be reduced with specific intervention programs, such as a mindfulness intervention, which have been shown to influence cognitive processes.

### Mindfulness and stereotype threat

Mindfulness describes the ability to stay in the present moment in an unbiased and non-judgmental way (e.g., Brown & Ryan, 2003). It is assumed that mindfulness practice involves attention regulation. In focus attention meditation (FAM), the attention is directed to an object or the breath; in open monitoring attention meditation (OMM), the meditator monitors the immediate perception but without directing the attention to one specific object (Dahl et al., 2015). Many meditation studies investigated the effect of more extended meditation practice, from 8 weeks upward. However, some studies also examined the effects of a mindfulness induction, which is a single and short session of mindfulness, as, for example, the task to eat two raisins mindfully (Schofield et al., 2015). In young adults, a short FAM, which requires focusing on a specific “object” like the breath during meditation and OMM meditation, includes an open observing of the things raising during meditation, affecting the processing in a cognitive control task in a different manner (Colzato et al., 2016). There was no difference in the so-called global-precedence effect (the faster processing of global vs. local features), but in the congruence effect (the observation that response time is longer if two stimuli were incongruent compared to two congruent stimuli), which was larger after OMM than after FAM. Many studies investigated the effect of mindfulness in adults, and a systematic review indicates that mindfulness interventions enhance executive functions, working memory, inhibition, and cognitive flexibility in children (Jansen et al., 2016).

To our best knowledge, only one study has investigated the effects of a mindfulness intervention on stereotype threat effects in detail: Weger et al. (2012) used a brief mindfulness intervention of 5 min to possibly alleviate the effects of stereotype threat (“why males are better than females in math”). Seventy-one female psychology students participated. They were required to complete two math tests as pre- and post-intervention tests and were randomly assigned to one of the following conditions: mindfulness induction (with versus without) and stereotype threat activation (activated versus non-activated). The results showed that women in the threat-activated condition performed significantly better after the mindfulness intervention than the controls, who did not receive a mindful intervention. This is in line with the assumption of Shapiro et al. (2006), who suggested that mindfulness meditation might reduce biased thinking because non-judgmental thinking is emphasized.

The underlying mechanism of a mindfulness intervention on a cognitive task following a stereotype threat activation could be its effect on the working memory. Working memory is a fundamental cognitive task, which is involved in higher cognitive tasks such as mental rotation (Hyun & Luck, 2007). However, if a stereotype threat effect appears, working memory might be affected because the stereotype threat effect produces stress that needs the same resources, which are used for working memory solving, e.g., a cognitive task (Beilock et al., 2007). However, if a mindfulness intervention can enhance working memory (Jha et al., 2019), it seems worth investigating if it can also counterbalance a stereotype threat in a cognitive task.

The main goal of the present study is to examine the effect of mindfulness on stereotype threat activation while solving a mental rotation test in early and late adolescence. In contrast to the study of Weger et al. (2012), who investigated the effects of a brief mindfulness intervention on stereotype threat only on women, the present study aims to analyze these effects for both male and female adolescents. Additionally, we aimed to investigate the effects of a stereotype threat activation and possible curbing effects of a brief mindfulness intervention on younger adolescents as well. For both age groups, effects of the mindfulness intervention on boys’ and girls’ performance independent of a stereotype threat should also be investigated. Therefore, male and female participants were included in the present study.

Mindfulness (Mesmer-Magnus et al., 2017) and stereotype threat (von Hippel et al., 2011) not only affect the performance, but are also associated with confidence or perceived performance. Furthermore, the perceived performance positively predicts the actual performance in a mental rotation task (Rahe & Jansen, 2021). Therefore, we not only analyzed the effects of stereotype threat activation and mindfulness on the mental rotation performance, but also on the perceived performance.

### Perceived abilities

Individuals’ beliefs about their abilities in certain activities can explain their performance, choice, and persistence in these activities (Wigfield & Eccles, 2000). This is the basis of the expectancy-value theory of achievement motivation. According to that theory, children form differentiated beliefs about their abilities in various activities, and these beliefs become more negative when children get older. In math, children’s beliefs about their abilities strongly predicted their grades (Wigfield & Eccles, 2000). When participants were asked about their confidence in their mental rotation task performance, a positive correlation with their actual performance was found (Cooke-Simpson & Voyer, 2007; Estes & Felker, 2012). Furthermore, men’s confidence ratings were more accurate than women’s ratings (Cooke-Simpson & Voyer, 2007), and men reported higher scores of confidence (Estes & Felker, 2012). In a study with 168 younger and older adolescents between 11 and 20 years, the results demonstrated that the psychometric mental rotation test performance could be predicted by participants’ gender, the perceived ability of stereotypically masculine activities, and their female gender stereotype beliefs (Rahe & Quaiser-Pohl, 2019).

Mindfulness meditation can enhance non-judgmental thinking and reduce biased thinking (Shapiro et al., 2006). A study showed a decrease in the effect of automatic stereotype-activated behavior toward older people after a mindfulness task (Djikic et al., 2008). A brief 10-min mindfulness audiotape could also reduce discrimination toward Black partners in the trust game (Lueke & Gibson, 2016). Regarding gender stereotype thinking, mindfulness is positively correlated to the motivation to respond without sexism (Gervais & Hoffman, 2013). Hence, it has to be investigated if gender stereotype beliefs can be affected by a brief mindfulness intervention.

A meta-analysis confirmed that trait mindfulness is positively correlated to confidence (Mesmer-Magnus et al., 2017). A study showed that self-confidence could be improved with eight mindfulness training sessions (Vala et al., 2016). Qualitative research revealed greater confidence regarding participants’ abilities to prevent burnout after a mindfulness class (Felton et al., 2015). Therefore, we aim to analyze the effects of a brief mindfulness intervention on adolescents’ perceived abilities.

### Goal of the study

We aim to analyze the effects of a short mindfulness induction in combination with a stereotype threat activation on the performance and the perceived performance in a mental rotation task as well as on gender-based stereotype beliefs. A stereotype threat activation should reduce girls’ (perceived) performance and a mindfulness induction should curb these reductions. For boys, reverse effects of a mindfulness induction on a possible stereotype lift effect—due to the activation—will also be examined.

### Research questions and hypotheses

1. Hypotheses regarding mental rotation performance: In line with previous studies (e.g., Titze et al., 2011), boys should outperform girls in the MRT, the gender difference should be more prominent in the older age group than the younger one (Rahe & Quaiser-Pohl, 2019), and older adolescents should show a better performance than younger ones (Titze et al., 2011). According to the study of Weger et al. (2012), we assume that a short mindfulness induction can improve mental rotation performance and reduce the effects of the stereotype threat on mental rotation performance in girls and young women. It has to be tested exploratively whether there is a stereotype lift effect in boys and young men and how mindfulness acts on this.
2. Hypotheses regarding gender-based stereotype beliefs: Because mindfulness practice is assumed to compensate biased thinking (Shapiro et al., 2006), we hypothesize that a short mindfulness intervention can reduce gender-based stereotype beliefs. Furthermore, the effects of gender, age, and stereotype threat were analyzed. Females should report stronger female stereotype beliefs, and males should report stronger male stereotype beliefs (Rahe & Quaiser-Pohl, 2019). These effects should be enhanced in the older age group. In the stereotype threat condition, male stereotype beliefs should be more pronounced than in those without a threat.
3. Hypotheses regarding the perceived abilities: We hypothesize that a short mindfulness intervention can strengthen self-related processes like the perceived abilities of feminine and masculine activities (Felton et al., 2015). Above that, gender and age differences, as well as the influence of a stereotype threat were examined. Females should rate their abilities in female activities higher than in male activities, while this effect is supposed to be reversed for males (Rahe & Quaiser-Pohl, 2019). This effect should be more pronounced in older adolescents Rahe & Quaiser-Pohl, 2021).

## Method

### Participants

In this study, 402 younger and older adolescents from four high schools in Germany participated. The group was divided into two age groups. The younger age group included 250 younger adolescents from grades 5, 6, and 7 (119 boys, mean age = 11.45 years, *SD* = 0.799 and 131 girls, mean age = 11.39 years, *SD* = 0.873). The older age group included 152 adolescents from grades 10, 11, and 12 (80 boys, mean age = 16.59 years, *SD* = 0.780 and 72 girls, mean age = 16.25 years, *SD* = 0.904). In the study of Weger et al. (2012), the difference within the stereotype-activated group between the mindful intervention and the non-mindful intervention was detected with a *d* = 0.92. The required sample size for girls in each age group was estimated using G*power (Faul et al., 2007). To ensure a sufficient sample size for an effect size of *d* = 0.92 (*f* = 0.46), a power of 0.95, and an alpha value of 0.05, a sample size of 64 girls and young women was estimated in each age group to detect a difference in the stereotype-activated group dependent on the mindfulness intervention.

All adolescents and their parents gave their written consent for participation. The experiment was conducted according to the ethical guidelines of the Helsinki Declaration. Ethical approval for this study was not required in accordance with the conditions outlined by the German Research Foundation, where research that carries no additional risk beyond daily activities does not require Research Ethics Board approval.

### Materials

#### Mental rotations test (MRT)

The paper–pencil MRT (according to Peters et al., 1995) consisted of 12 items, each with five cube figures rotated in depth. For each item, one cube figure on the left (target item) had to be compared to four test objects (sample items) on the right. Two of the four sample stimuli were identical to the target item and had to be crossed out. Both remaining sample stimuli were mirrored images of the target item. One point per item was given only if both correct figures were crossed out and no mirrored image was marked. Thus, a total of 12 points could be achieved.

#### Stereotype beliefs

For stereotype beliefs, a questionnaire (according to Moè et al., 2009) consisted of 11 activities: 5 of these were supposed to be male stereotyped (e.g., finding a way on a map), 5 female stereotyped (e.g., learning a new language), and 1 was a neutral activity (solving a crossword puzzle). For each activity, adolescents were asked whether they thought males or females were better performing this activity. The seven-point answering scale ranged from three (males are better) on the left to three (females are better) on the right, including zero as a neutral answering option. For data entry, 3, 2, and 1 on the left were transformed into −3, −2, and −1. One-sample *t* tests revealed that the mean scores of stereotyped male activities significantly differed from the neutral point to the negative pole (all *p* < 0.001), and stereotyped female activities significantly differed from the neutral to the positive pole (all *p* < 0.009). The neutral activity did not differ from the neutral point (*p* = 0.430).

A factor analysis of the ten gender-stereotyped activities confirmed two factors of male stereotype beliefs and female stereotype beliefs (KMO = 0.636, *p* < 0.001). Mean scores were computed for both new variables; for male stereotype beliefs, the mean scores were multiplied with − 1 to ensure the same scale as for female stereotype beliefs, with higher scores indicating more pronounced stereotype beliefs.

#### Perceived ability of masculine and feminine activities

For the perceived ability of masculine and feminine activities, the same 11 activities were listed. Adolescents were asked to evaluate their performance in these activities on a six-point scale ranging from one (very good) to six (very bad). A factor analysis confirmed two factors of the perceived ability of masculine and feminine activities (KMO = 0.704, *p* < 0.001). Two variables were computed by calculating mean scores of the perceived abilities of the five masculine and, respectively, the five feminine activities. Both mean scores were then recoded, ensuring that higher scores indicating higher abilities.

### Procedure

All adolescents were tested in their classes. All classes were randomly assigned within one grade to one of four conditions with or without a mindfulness induction and with or without stereotype threat activation. Before testing, each adolescent received two candies. In the condition without mindfulness induction, the participants were given 5 min to eat the candies with an instruction to be quiet and not to speak with anybody. In the mindfulness condition, adolescents heard a 5-min recorded instruction to eat the candies while being mindful about it (Jansen et al., 2019). Participants should handle the candies as if they have never seen them before. They should look at each part carefully, smell them, weigh them in their hand, place it in their mouths, and finally, chew slowly and carefully. Afterward, adolescents in the stereotype threat condition heard a short explanation that this study was conducted to learn more about why males are better in mental rotation. Adolescents in the non-stereotyped threat condition did not receive any explanation. The MRT was then distributed to all participants. Two practice items were solved and discussed in the whole class to ensure that all participants understood the task. Afterward, 12 items had to be solved in 3 min, which correspond to half of the original MRT (Peters et al., 1995). After this, the adolescents filled out a demographic questionnaire with age and gender and the two questionnaires about their stereotype beliefs and their perceived ability of masculine and feminine activities.

### Statistical analysis

First, a *t* test with the dependent variable stereotype beliefs (item: “rotating objects in one’s mind”) and the independent variable stereotype threat condition (with/without) was calculated to check the manipulation of the stereotype threat.

Secondly, a univariate analysis of variance with the dependent variable MRT score and the between-subject factors gender (boys, girls), age group (younger AG, older AG), mindfulness intervention (with, without), and stereotype activation (with, without) was conducted. All post hoc tests were Bonferroni corrected with *p* divided by the number of *t* tests.

Thirdly, two repeated measurements were performed regarding (a) the stereotype belief task with the within-subject factor belief (male versus female) and the between-subject factors mindfulness (with and without), age groups and gender, and (b) the perceived ability with the within-subjects factor abilities (masculine versus feminine activities) and the between-subject factors mindfulness (with, without), stereotype threat (with, without), age group, and gender. All post hoc tests were Bonferroni corrected with *p* divided by the number of *t* tests.

## Results

For a manipulation check of the stereotype threat condition, a *t* test was calculated to compare means in the stereotype beliefs variable (“rotating objects in one’s mind”) between both stereotype threat conditions. Adolescents in the stereotype threat condition (*M* = 0.91, *SD* = 1.05) had stronger beliefs (*t*(393) = 4.815, *p* < 0.001) that rotating objects in one’s mind was a masculine ability than participants who did not receive the stereotype threat (*M* = 0.41, *SD* = 1.02).

### Mental rotation performance

**Fig. 1:**
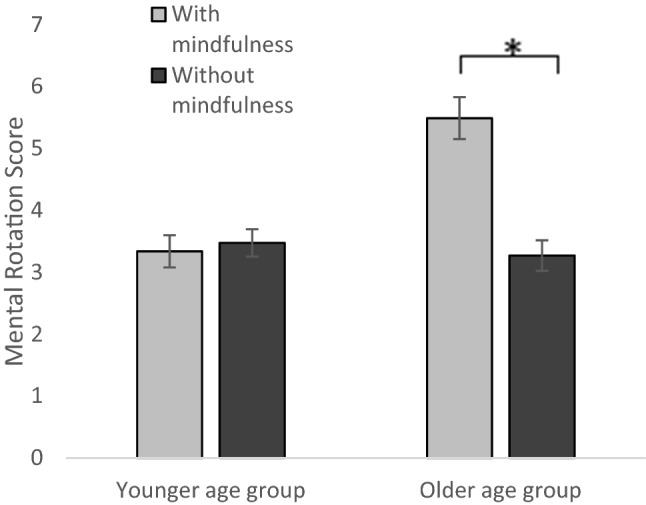
Mean mental rotation scores dependent on age group and mindfulness intervention. Error bars indicate *SE*. Note: younger age group = grades 5–7, older age group = grades 10–12. The mindfulness intervention increases performance only for the older age group

A univariate analysis of variance with the dependent variable MRT score and the between-subject factors gender (boys, girls), age group (younger AG, older AG), mindfulness intervention (with, without) and stereotype activation (with, without) was conducted. It showed three main effects of age group, *F*(1, 386) = 10.09, *p* = 0.002, partial *η*^2^ = 0.025, mindfulness, *F*(1, 386) = 5.15, *p* = 0.024, partial *η*^2^ = 0.013, and gender *F*(1, 386) = 24.35, *p* < 0.001, partial *η*^2^ = 0.059. Older adolescents (*M* = 4.51, *SD* = 3.05) demonstrated a better performance than the younger adolescents (*M* = 3.41, *SD* = 2.66), boys (*M* = 4.59, *SD* = 3.06) showed a better performance than girls (*M* = 3.08, *SD* = 2.43) and participants who received the mindfulness intervention (*M* = 4.25, *SD* = 2.12) performed better than those without mindfulness intervention (*M* = 3.40, *SD* = 2.52). Furthermore, there were two significant two-way interactions between age group and mindfulness, *F*(1, 386) = 5.46, *p* = 0.020, partial *η*^2^ = 0.014 and age group and gender, *F*(1, 386) = 5.99, *p* = 0.015, partial *η*^2^ = 0.015. Whereas there was no significant difference between both mindfulness conditions in the younger age group, the mindfulness group (*M* = 5.49, *SD* = 3.13) in the older age group showed a better mental rotation performance than the group that did not receive an intervention (*M* = 3.27, *SD* = 2.47), *t*(150) = 4.768, *p* < 0.001, *d* = 0.779, see Fig. 1.

Regarding the interaction between gender and age group, there was no significant gender difference in the younger age group, but a large difference in the older age group, *t*(150) = 6.24, *p* < 0.001, *d* = 1.019. Boys (*M* = 5.82, *SD* = 2.95) performed better than girls (*M* = 3.05, *SD* = 2.46). All post hoc tests were Bonferroni corrected with *p* divided by the number of *t* tests. No other main or interaction effects reached significance.

### Stereotype beliefs

**Fig. 2:**
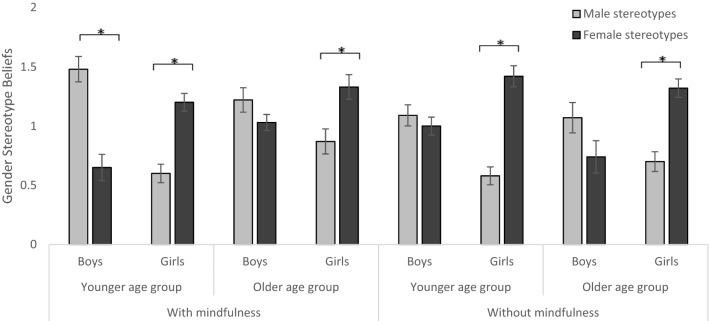
Gender stereotype beliefs dependent on age group, gender, and mindfulness intervention. Error bars indicate *SE*. Note: Younger age group = grades 5–7, older age group = grades 10–12

Regarding the stereotype beliefs, there was a main effect of stereotype beliefs, *F*(1, 383) = 11.30, *p* < 0.001, partial *η*^2^ = 0.029, a significant interaction of stereotype beliefs and gender, *F*(1, 383) = 154.66, *p* < 0.001, partial *η*^2^ = 0.288, a significant interaction of stereotype beliefs and mindfulness, *F*(1, 383) = 11.28, *p* < 0.001, partial *η*^2^ = 0.029, a three-way interaction of stereotype beliefs, age group, and gender *F*(1, 383) = 4.90, *p* = 0.027, partial *η*^2^ = 0.013, a three-way interaction of stereotype beliefs, age group, and mindfulness, *F*(1, 383) = 7.73, *p* = 0.006, partial *η*^2^ = 0.020, a significant three-way interaction of age group, mindfulness, and stereotype threat activation, *F*(1, 383) = 8.26, *p* = 0.004, partial *η*^2^ = 0.021, a significant four-way interaction of stereotype beliefs, age group, gender, and mindfulness, *F*(1, 383) = 6.17, *p* = 0.013, partial *η*^2^ = 0.016, and a significant four-way interaction of stereotype beliefs, gender, mindfulness, and stereotype threat activation, *F*(1, 383) = 3.93, *p* = 0.048, partial *η*^2^ = 0.010. The four-way interaction of stereotype beliefs, age group, gender, and mindfulness (Fig. 2) shows that young boys who received a mindfulness intervention had stronger male (*M* = 1.48, *SD* = 0.75) than female (*M* = 0.64, *SD* = 0.78) stereotype beliefs, *t*(49) = 5.48, *p* < 0.001, *d* = 0.775. No differences were found for younger and older boys in the condition without mindfulness intervention, and for older boys in the mindfulness condition. In contrast, all girls had stronger female than male stereotype beliefs (all *p* < 0.001). All post hoc tests were Bonferroni corrected with *p* divided by the number of *t* tests.

**Fig. 3:**
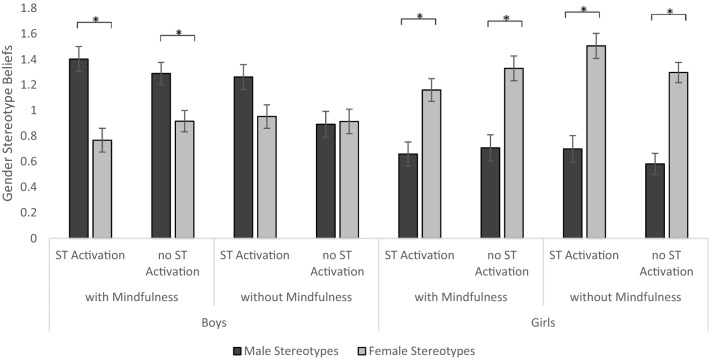
Gender stereotype beliefs dependent on gender, mindfulness intervention, and stereotype threat (ST) activation. Error bars indicate *SE*

Regarding the significant four-way interaction of stereotype beliefs, gender, mindfulness, and stereotype threat activation (Fig. 3), male stereotype beliefs were significantly more substantial than female stereotype beliefs in boys with a mindfulness intervention (all *p*s < 0.003) but not without the intervention. In girls, this effect is reversed: their female stereotype beliefs were significantly more substantial than their male stereotype beliefs independent of the mindfulness intervention (all *p*s < 0.001). Above that, it seems that a stereotype threat activation increases the difference between male and female stereotype beliefs in boys, while this difference in girls’ beliefs is unaffected by stereotype threat.

### The ability of masculine and feminine activities

**Fig. 4:**
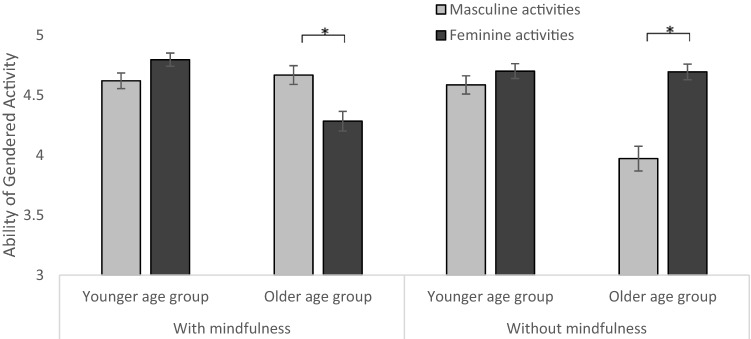
Ability of masculine and feminine activities dependent on age group and mindfulness intervention. Error bars indicate *SE*. Note: younger age group = grades 5–7, older age group = grades 10–12

Concerning the perceived ability of masculine and feminine activities, the results showed two main effects of ability, *F*(1, 386) = 11.43, *p* < 0.001, partial *η*^2^ = 0.029 and age group, *F*(1, 386) = 18.13, *p* < 0.001, partial *η*^2^ = 0.045, a two-way interaction of ability and gender, *F*(1, 386) = 146.45, *p* < 0.001, partial *η*^2^ = 0.275, a two-way interaction of ability and mindfulness *F*(1, 386) = 12.66, *p* < 0.001, partial *η*^2^ = 0.033, a three-way interaction of ability, mindfulness, and age group, *F*(1, 386) = 9.04, *p* = 0.003, partial *η*^2^ = 0.023 (see Fig. 4), and a three-way interaction of mindfulness, age group, and stereotype activation, *F*(1, 386) = 5.66, *p* = 0.018, partial *η*^2^ = 0.014. Concerning the three-way interaction of ability, mindfulness, and age group, older participants in the mindfulness condition reported better abilities in masculine than feminine activities, *t*(85) = 3.11, *p* = 0.003, *d* = 0.335, whereas the opposite result was found for older participants who did not receive a mindfulness intervention, *t*(66) = -5.92, *p* < 0.001, *d* = 0.723.

## Discussion

Regarding our first hypothesis, our results revealed that older adolescents outperformed younger adolescents in their mental rotation performance and that the gender difference in favor of boys could be detected, mainly in the older age group. The short mindfulness induction ameliorates the mental rotation performance for the older age group, but this effect was independent of the stereotype threat activation. Concerning our second hypotheses, the results did not demonstrate a reduced stereotype bias, but a stereotype lift effect for older boys after a short mindfulness intervention regarding their male beliefs. Furthermore, older participants reported better abilities in male activities than female activities after the mindfulness intervention.

### The influence of a mindfulness induction in mental rotation and self-related processes

Although the stereotype threat was activated, we did not find an effect on the performance in the mental rotation task. For this, we could not analyze an effect regarding reducing the stereotype threat through mindfulness induction. However, we found an enhancing effect of the short mindfulness induction in the mental rotation performance for the older age group. It is assumed that through mindfulness induction, an attention allocation was induced, and mind-wandering was diminished. This is in line with the mechanistic models of mindfulness, which emphasize the importance of attentional and self-referential processes (e.g., Hölzel et al., 2011). Another reason might be reduced anxiety or arousal, which is following other studies demonstrating a reduction of negative thoughts after a short mindfulness intervention (Thompson et al., 2021).

Regarding the effect of our short mindfulness induction on stereotype beliefs and the ability of masculine and feminine activities, the short intervention has an effect only in the older age group in that masculine attributes were lifted, either only for boys or girls and boys. This result contrasts with the assumption of Shapiro et al. (2006), who found that mindfulness practice could compensate biased thinking (Shapiro et al., 2006). In the present study, it rather emphasizes younger boys’ gender-stereotyped beliefs. Similar results were found by Gebauer et al. (2018) demonstrating that a 15-min loving-kindness meditation exacerbated self-centrality in meditation-relevant domains. This means that a short meditation might not diminish some aspects of the self like self-related biased thinking, but emphasize it. No gender differences were investigated by Gebauer et al. (2018), thus the stereotype lift effect in boys was a new result and should be investigated in more detail. It is well known that boys show higher self-esteem in several domains in adolescence (Quatman & Watson, 2001). The short mindfulness intervention seems to emphasize the male attributes in gender beliefs for the younger group. The results of the different evaluations from older boys and girls in their abilities in feminine and masculine activities are also new and need further investigation. Next to the investigation of the effect of a mindfulness intervention, the results give insight into gender difference in mental rotation performance during adolescence and the possible relating factor of stereotype threat.

### Gender differences in mental rotation performance during adolescence

The better performance of boys was evident in the older age group and only small and not significant in the younger age group. This result adds to the ongoing debate on gender differences in mental rotation during adolescence. In some studies, better mental rotation performance of boys in the MRT is already present within the age range of ten years (Titze et al., 2010) and exists in adolescence (Titze et al., 2011). On the other hand, no gender difference could be detected for boys and girls between 12 and 17 years (Jansen et al., 2018). Another study provides evidence that gender differences in the MRT were not significant in a younger adolescent age group (up to 15 years), but appeared in adolescents older than 15 years (Rahe & Quaiser-Pohl, 2019). All these results hint that the phenomenon of possible gender differences in a psychometric mental rotation test is not clarified. Biological and social reasons seem to be more important than age. However, in a study with children between 9–14 years, a relationship between the hormonal levels and any measure of mental rotation performance could not be detected (Quaiser-Pohl et al., 2016), and further relevant studies on the relationship between hormones and mental rotation performance in adolescence are missing. In contrast to the missing studies regarding the possible causing effects on mental rotation performance in adolescence, social influencing factors are investigated in more detail, as, for example, the attendance of a specific type of schools, for example, single-gender schools (Titze et al., 2011), schools with an augmented physical activity during the school day (Jansen et al., 2018), or the attendance of subjects of natural science (Moè, 2016b). Furthermore, and at least in primary school-aged children, socioeconomic status modifies sex differences in spatial skills (Levine et al., 2005).

Moreover, stereotype threat seemed to be one explaining factor. For fourth graders, it was demonstrated that the gender effect in mental rotation is affected by stereotype threat and stereotype lift from the very beginning of its occurrence (Neuburger et al., 2012). In adults, implicit gender spatial stereotyping also exists (Guizzo et al., 2019). To the best of our knowledge, no study has investigated the possible stereotype threat vs. lift effects on mental rotation in adolescence. In this study, a stereotype threat, respectively, a stereotype lift effect could not be evoked. We cannot evaluate if this is specific to the study’s design or a general phenomenon in mental rotation in adolescence; relevant studies are missing. One explanation might be that the participants in the study presented here did not identify themselves with the stereotyped domain, which means that the performance in this domain, mental rotation, is not self-relevant. For math, it has been demonstrated that stereotype threat effects were most prominent among women who were moderately identified with math (Nguyen & Ryan, 2008). Another explanation for the missing stereotype threat or lift effects might be that the mental rotation task could be a stereotype trigger itself, so it might be helpful to manipulate the instruction or the rotational objects to reduce the “stereotype load” of the mental rotation test.

### Limitation

In this study, the influence of a short mindfulness induction has been investigated on a cognitive task and self-related processes in adolescents. Even though many young people participated, the study is limited by some aspects. First, the performance in the mental rotation task was not retrieved before the interventions. Differences in the pre-test measurement cannot be excluded but might be disregarded due to the high number of participants. Furthermore, only half of the MRT of Peters et al. (1995) has been used, which is in line with studies of, e.g., Meneghetti et al. (2011) applying a short version of the MRT of ten items in each test session. Another limitation is the short mindfulness induction. Even though this task has been used before and the effects of a single session of mindfulness are investigated, we must keep in mind that mindfulness primarily works through daily practice over a longer time. This is important to keep in mind for further study designs on effects of mindfulness on stereotype threat in cognitive research (e.g., see for the claim of well-designed studies, Van Dam et al., 2018). Furthermore, answers to the perceived ability questionnaire could have been influenced by the answers to the stereotype belief questionnaire. Finally, we randomized whole classes to the conditions but not individual children and adolescents.

## Conclusion

The present study demonstrated that a short mindfulness induction could enhance performance in a stereotypically masculine cognitive task. This was independent of participants’ gender but appeared only in the older age group. The same training also strengthened younger boys’ male stereotype beliefs and adolescents’ perceived ability of masculine activities, but only for the older age group. From a practical point of view, this suggests the use of such training. From a theoretical point of view, this study offers new ideas to investigate the enhancement of specific cognitive processes through a short mindfulness training in more detail as well as the underlying mechanism.

## Data Availability

The datasets generated during and/or analyzed during the current study are available from the corresponding author on reasonable request.
